# Prediction of Human's Ability in Sound Localization Based on the Statistical Properties of Spike Trains along the Brainstem Auditory Pathway

**DOI:** 10.1155/2014/575716

**Published:** 2014-03-31

**Authors:** Ram Krips, Miriam Furst

**Affiliations:** The School of Electrical Engineering, Faculty of Engineering, Tel Aviv University, 69978 Tel Aviv, Israel

## Abstract

The minimum audible angle test which is commonly used for evaluating human localization ability depends on interaural time delay, interaural level differences, and spectral information about the acoustic stimulus. These physical properties are estimated at different stages along the brainstem auditory pathway. The interaural time delay is ambiguous at certain frequencies, thus confusion arises as to the source of these frequencies. It is assumed that in a typical minimum audible angle experiment, the brain acts as an unbiased optimal estimator and thus the human performance can be obtained by deriving optimal lower bounds. Two types of lower bounds are tested: the Cramer-Rao and the Barankin. The Cramer-Rao bound only takes into account the approximation of the true direction of the stimulus; the Barankin bound considers other possible directions that arise from the ambiguous phase information. These lower bounds are derived at the output of the auditory nerve and of the superior olivary complex where binaural cues are estimated. An agreement between human experimental data was obtained only when the superior olivary complex was considered and the Barankin lower bound was used. This result suggests that sound localization is estimated by the auditory nuclei using ambiguous binaural information.

## 1. Introduction

Adrian's classic research on neural activity [1] presented three essential observations which are as relevant today as they were when he first introduced them: (1) as individual neurons produce action potential which propagate through the brain, the information of the neural activity is encoded by spiking events; (2) the rate of the spikes is dependent upon the external stimuli that drives the neural cell; and (3) there is an adaptation mechanism that adjusts the cell response; that is, the neural activity is reduced for constant stimuli. Any model that purports to characterize a neural activity must take into account these basic principles.

In this study we refer to auditory systems in which irregular neuronal activity was demonstrated during* in vivo* recordings [2].* In vivo* observations have also shown that a specific neuron might respond with a single spike or several spikes to a given stimuli as shown in [2]. Kiang's [2] observation is not in agreement with that of Adrian [1], who suggested that the stimuli information is coded by the average rate of the neural response. This contradiction raises the possibility that the timing of the spikes relative to the stimulus should be considered as well.

The origin of the stochastic activity of neurons is poorly understood. This activity results in both intrinsic noise sources that generate stochastic behavior on the level of the neuronal dynamics and extrinsic sources that arise from network effects and synaptic transmission [3]. Another source of noise that is specific to neurons arises from the finite number of ion channels in a neuronal membrane patch [4, 5].

There are a number of different ways that have emerged to describe the stochastic properties of neural activity. One possible approach relates to the train of spikes as a stochastic point process. For example, in their earlier studies, Alaoglu and Smith [6] and Rodieck et al. [7] suggested that the spontaneous activity of the cochlear nucleus can be described as a homogeneous Poisson process. Further investigations of the auditory system described the neural response as a nonhomogeneous Poisson point process (NHPP) whose instantaneous rate depends on the input stimuli [8, 9].

A meaningful characterization of neural activity can be derived by using stochastic properties in order to predict human performance. Up to the 19th century, when medical science was still in its infancy and the concept of neural activity was unknown, the only method of understanding and researching the brain was through a black-box approach based on psychoacoustical experiments. While these psychoacoustical experiments provided valuable information, they were regarded as limited since they only produced qualitative information. It was argued that the activities and the contents of the mind could not be measured and therefore could not be objective. This view began to change in the early 1800s when Ernst Weber (1795–1878) demonstrated two measures for quantifying psychological data that he obtained from testing subjects psychoacoustically: (1) the two-point threshold, in which the smallest distance noticeable to touch at various parts of the body is measured, and (2) the just-noticeable difference (JND), in which the smallest difference in weight a person is capable of distinguishing is measured.

In the mid-20th century, several classes of standard adaptive tests for psychoacoustic measurements were introduced for evaluating auditory resolution [10–12]. These measurements are used for comparing the relationship between prediction of neural models and psychoacoustical performances. In such psychoacoustical tests, subjects are asked to distinguish between close values of one of the signal's parameters, such as the signal's frequency or level in monaural stimulation, and the interaural level difference (ILD), or the interaural time difference (ITD) in binaural stimulation. The results of such experiments are the JND of the investigated parameter. Such experiments have been repeatedly performed and reported in the literature (e.g., [13–20]).

Comparing the behavioural JND and the neural activity is possible if one assumes that the neural system estimates the measured parameters. Siebert [21, 22] obtained such a comparison when the JND of a single tone's frequency and level was compared to the neural activity of the auditory nerve. Siebert's findings were based on the assumption that the auditory nerve (AN) response behaves as a NHPP, and the brain acts as an unbiased optimal estimator of the physical parameters. Thus, the JND is equal to the standard deviation of the estimated parameter and can be derived by lower bounds such as the Cramer-Rao lower bound. Heinz et al. [23, 24] generalized Siebert's results to a larger range of frequencies and levels. Colburn and his colleagues [25–29] obtained similar evaluations for binaural signals, where the JND of ITD and ILD was compared to the neural activity of the auditory nerves of both ears.

This approach was extended to analyze brainstem nuclei such as the superior olivary complex (SOC) and the inferior colliculus (IC). These nuclei receive inputs from both ears, integrate the information, and send it by means of neural spike trains to the upper nuclei in the auditory pathway [30–36].

The neural cells in the SOC and IC are frequently described as coincidence detector (CD) cells. These cells receive independent excitatory and inhibitory inputs and generate a spike if the number of excitatory inputs exceeds the number of inhibitory inputs by a known number during a short interval. Krips and Furst [37] showed that the CD cells behave as NHPP if their inputs are NHPP. Therefore, the JND of the binaural parameters such as ITD and ILD, which are presumably estimated at the level of the SOC or IC, can be derived on the basis of the CD cell outputs [38].

Two main types of CD cells are identified in the brainstem auditory pathway: excitatory-excitatory (EE) cells and excitatory-inhibitory (EI) cells. EE cells receive excitatory inputs from both (right and left) anteroventral cochlear nuclei (AVCN) and they fire when both inputs are received within a time interval of less than 50 *μ*sec [36, 39–41]. These types of cells are sensitive mainly to ITD. EI cells, on the other hand, are sensitive to the balance of intensity at the ears because the excitation, due to ipsilateral stimuli, is reduced by increasing levels of contralateral stimuli [40, 42–45].

The human ability to localize sound depends on ITD, ILD, and spectral information of the acoustic stimulus. The goal of this paper is to test whether the prediction of human performance in this task is possible from the AN response or whether the processing of higher auditory brainstem nuclei is required. We compare the prediction of human performance based on the stochastic properties of the spike trains at the level of the auditory nerve and at the level of the SOC.

## 2. Minimum Audible Angle

The minimum audible angle (MAA) test is a common means of evaluating human localization ability. In this test, two successive signals from different directions are aimed at a listener. The order of the two signals is random. The listener is instructed to indicate the direction of the two signals relative to each other. For example, in the horizontal plane, the subject is asked if the signal moved from right to left, or vice versa.

**Figure 1:**
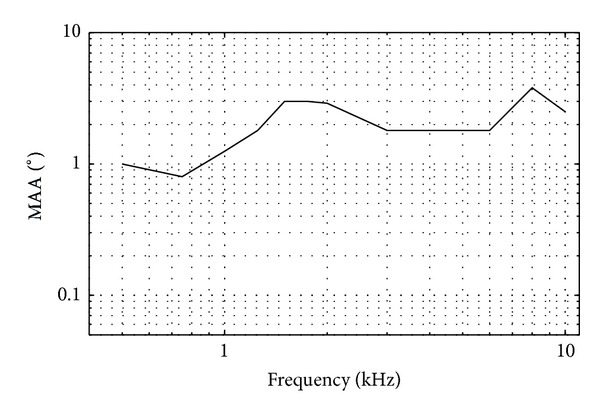
MAA experimental results as a function of frequency for a reference azimuth of 0°, (redrawn from [1]).

MAA experiments have been conducted with various experimental setups and testing procedures for different stimuli conditions [11, 46–50]. For a single-tone MAA in the horizontal plane, Mills' measurements [11] have become the generally accepted standard. MAA as a function of frequency at an azimuth of 0° is redrawn from Mills' [11] measurements in Figure 1. The MAA exhibits the following properties: (1) an increase of MAA as a function of frequency above 1 kHz and (2) an oscillatory behavior as a function of frequency with local maxima at about 1.5 and 8 kHz.

In a typical MAA experiment, the audio signal *S*(*t*, *θ*) enters both ears from a direction *θ* relative to the nose. The incoming sounds to each ear are transformed as a function of the shape and size of the head, torso, and the pinna of the outer ears. These anatomical features are known as the head-related-transfer-function (HRTF) that can be measured and synthesized in the form of linear time-invariant filters.

**Figure 2:**
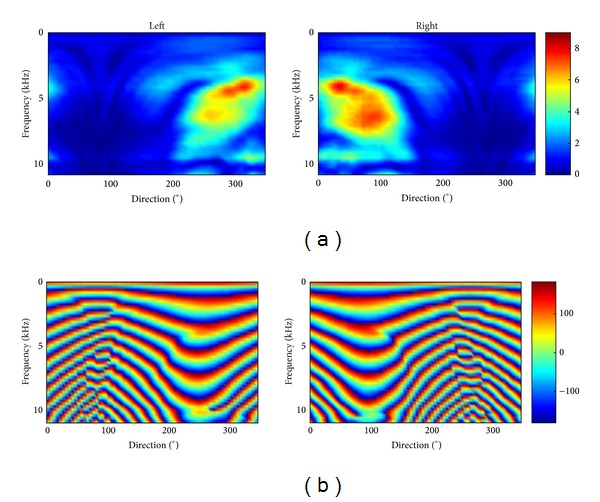
A sample HRTF gain in dB (a) and phase in degrees (b) as a function of azimuth and frequency for 0° elevation.

In Figure 2, typical right and left HRTFs for an elevation of 0° are presented. They were obtained from Knowles Electronic Manikin for Acoustics Research (KEMAR) [51]. Both gain (Figure 2(a)) and phase (Figure 2(b)) are demonstrated in Figure 2 by color-coded scales. They are plotted as a function of both frequency (*f*) and direction (*θ*). The maximum gain for the right HRTF is obtained at high frequencies when the speaker is located in front of the right ear, that is, a direction of 90°. Similarly, maximum gain for the left HRTF is obtained when the speaker is located in front of the left ear, which corresponds to a direction of 270°. At frequencies above 1 kHz, the phase becomes ambiguous since different directions yield similar phases.

Formally, the signals that are conveyed to the left and right cochleae are

(1) SL(t,θ)=S(t,θ)∗HRIRL(t,θ),SR(t,θ)=S(t,θ)∗HRIRR(t,θ),

where ∗ represents a convolution and HRIR_L_(*t*, *θ*) and HRIR_R_(*t*, *θ*) are the left and right head-related impulse responses, respectively.

In this study, we refer only to signals that are composed of simple tones, that is, *S*(*t*) = *A*sin⁡(2*πft*), where *A* is the signal amplitude and *f* represents its frequency. The effects of the HRTF on such a signal are phase shifts and amplitude alterations that yield

(2) SL(t,θ)=AL(θ)sin⁡(2πft+φL(θ)),SR(t,θ)=AR(θ)sin⁡(2πft+φR(θ)).

Therefore, the resulting interaural differences are a phase difference (IPD) that is obtained by

(3) $$\begin{array}{c} \text{IPD}\left(\theta \right)=\varphi_{\text{R}}\left(\theta \right)-\varphi_{\text{L}}\left(\theta \right), \end{array}$$

which corresponds to ITD by

(4) $$\begin{array}{c} \text{ITD}\left(\theta \right)=\frac{\text{IPD}\left(\theta \right)}{2\pi f}, \end{array}$$

and interaural level difference (ILD) in dB is given by

(5) $$\begin{array}{c} \text{ILD}\left(\theta \right)=20{\;\log}_{10}\left(\frac{A_{\text{R}}\left(\theta \right)}{A_{\text{L}}\left(\theta \right)}\right)=20{\;\log}_{10}\left(\delta \right), \end{array}$$

where *δ* = *A*_R_(*θ*)/*A*_L_(*θ*).

## 3. Estimating MAA on the Basis of the Stochastic Properties of Neural Spike Trains

We assume that during an MAA experiment, the brain's task is to estimate *θ*. The resultant unbiased estimator is $\hat{\theta }$, which yields

(6) $$\begin{array}{c} E\left[\hat{\theta }\;|\;\theta^{*}\right]=\theta^{*}, \end{array}$$

where θ* is the true direction of the incoming signal. Generally, in a psychoacoustical JND experiment, the yielded JND value is obtained when *d*′ = 1, where in an MAA experiment

(7) $$\begin{array}{c} d^{\prime }=\frac{E\left[\hat{\theta }\;|\;\theta^{*}\right]-E\left[\hat{\theta }\;|\;\left(\theta^{*}+\Delta \theta \right)\right]}{\text{std}\left(\hat{\theta }\;|\;\theta^{*}\right)}=\frac{\Delta \theta }{\text{std}\left(\hat{\theta }\;|\;\theta^{*}\right)}. \end{array}$$

Therefore, *d*′ = 1, yields the relations:

(8) $$\begin{array}{c} \Delta \theta =\text{MAA}=\text{std}\left(\hat{\theta }\;|\;\theta^{*}\right). \end{array}$$

In an optimal system, the standard deviation of the estimator, $\text{std}\left(\hat{\theta }|\theta^{*}\right)$, can be obtained by the Cramer-Rao lower bound (CRLB). This bound is achievable when the estimator uses information from the vicinity of the true value, θ*. However, in estimating the direction of sine waves when their phase information is ambiguous (Figure 2), the brain might consider different directions as the true ones. For example, when a continuous 2 kHz tone reaches both ears from either one of the sides or from the front of the head, the produced ITD in all cases will be 0. Thus, when the signal is coming from either of those directions, an optimal estimator can choose any of those possibilities. Since the Barankin lower bound (BLB) [52] takes into account different possible values of the estimated parameter other than those located in the proximity of the true one, the BLB might be a better choice in deriving a lower bound of $\text{std}\left(\hat{\theta }|\theta^{*}\right)$.

Let us define CRLB(θ*) and BLB(θ*) as the CRLB and the BLB of θ*, respectively. In general,

(9) $$\begin{array}{c} \text{MAA}=\text{std}\left(\hat{\theta }\;|\;\theta^{*}\right)\geq \text{BLB}\left(\theta^{*}\right)\geq \text{CRLB}\left(\theta^{*}\right). \end{array}$$

In order to derive both CRLB(θ*) and BLB(θ*), one should consider the probability density function of the estimator $\hat{\theta }|\theta^{*}$. The stochastic properties of the estimator $\hat{\theta }|\theta^{*}$ are initiated by the probabilistic behavior of the neural spike trains along the auditory pathway. Thus, the lower bounds can be derived from the probability density function of the neural spike trains.

The stochastic properties of the neural spike are described by the probability of getting *N* successive spikes during *T* seconds at the time instances {*t*_1_,…, *t*_*N*_} following an acoustic stimulus. As was stated earlier [8, 9], this behavior can be described as NHPP; therefore,

(10) $$\begin{array}{c} p\left(t_{1},\ldots ,t_{N}\right)=\frac{1}{N!}\overset{N}{\underset{n=1}{\prod }}\lambda \left(t_{n},\underline{\Theta }\right)\exp\left\{-\overset{T}{\underset{0}{\int }}\lambda \left(t,\underline{\Theta }\right)dt\right\}, \end{array}$$

where $\lambda \left(t,\underline{\Theta }\right)$ is the instantaneous rate of the neural point process and $\underline{\Theta }$ is a vector that includes all the physical parameters of the audio signal. In this study, since we relate to MAA, we choose $\underline{\Theta }=\theta$ as the direction of the incoming signal.

In NHPP, both lower bounds, CRLB and BLB, depend only on the instantaneous rate. The CRLB for a NHPP was derived by Bar David [53] and is given by

(11) $$\begin{array}{c} \text{CRLB}\left(\theta^{*}\right)={\left\{\overset{T}{\underset{0}{\int }}\frac{1}{\lambda \left(t,\theta^{*}\right)}{\left[{\frac{\partial \lambda \left(t,\theta \right)}{\partial \theta }|}_{\theta =\theta^{*}}\right]}^{2}dt\right\}}^{-1/2}. \end{array}$$

For deriving BLB, we define a vector of *L* that includes all the nontrue but possible values $\underline{\Phi }=\left[\theta_{1},\ldots ,\theta_{L}\right]$. In [37] the BLB was derived for an NHPP which is given by

(12) BLB(θ∗)=CRLB(θ∗)+(Φ_−CRLB(θ∗)·A_)Δ−1×(Φ_−CRLB(θ∗)·A_)T,

where $\underline{A}=\left[A_{1},\ldots ,A_{L}\right]$ is a vector of length *L*, when each *A*_*l*_ is given by

(13) $$\begin{array}{c} A_{l}=\overset{T}{\underset{0}{\int }}{\left[\frac{\lambda \left(t,\theta_{l}\right)}{\lambda \left(t,\theta^{*}\right)}-1\right]\cdot \frac{\partial \lambda \left(t,\theta \right)}{\partial \theta }|}_{\theta =\theta^{*}}dt. \end{array}$$

The matrix $\Delta =B-{\underline{A}}^{T}\text{CRLB}\left(\theta^{*}\right)\underline{A}$, where *B* is a symmetric matrix whose size is *L* × *L*. Each element in the matrix *B* is obtained by

(14) Bij=exp⁡⁡(∫0T[−λ(t,θi)−λ(t,θj)+λ(t,θ∗)+λ(t,θi)λ(t,θj)λ(t,θ∗)]dt).

The vector $\underline{\Phi }$ is essential in BLB derivation. If the size of the vector is predetermined, the actual values *θ*_1_,…, *θ*_*L*_ can be obtained by deriving BLB for all the possibilities. The* L* directions that yield the maximum BLB are then chosen for vector $\underline{\Phi }$. Such a straightforward approach is a time-consuming process that requires calculating enormous number of possible sets. For example, for *L* = 4 with a resolution of 1°, there are 360^4^ sets to consider. In order to reduce the number of calculations, a two-stage procedure was designed. In the first stage, for every frequency, BLB predicted MAAs based on a single ambiguity. In a 1° resolution, a total of 360 BLB derivations were obtained. In the second stage, for every frequency, the number of ambiguous directions (*L*) was defined and the vector [*θ*_1_,…, *θ*_*L*_] of the ambiguous directions was chosen according to directions that yielded maximum MAA in the first stage.

## 4. MAA Prediction Based on Auditory Nerve Response

Since the auditory nerve (AN) is the initial stage in the auditory neural pathway, we first tested the prediction of MAA on the basis of its response.

There are about 30,000 AN fibers that innervate each ear. The different location of each fiber's attachment on the cochlear partition determines its frequency sensitivity since each point along the cochlea has a different resonance frequency.

The auditory nerve's instantaneous rate (IR) for a simple tone stimulus *s*(*t*) = *A* · sin⁡(2*πft* + *φ*) is commonly expressed with an exponential function [21, 24, 25, 29, 38] which is obtained by

(15) λAN(t)=γ(f,A)·exp⁡{γ(f,A)·B(f)·sin(2πft+φ+ϕ(f))}.

Generally, *γ*(*f*, *A*) is a nonlinear function of both the level and frequency of the stimulus. Its minimum value equals the fiber's spontaneous rate while its maximum value is equal to the fiber's saturation rate. For stimuli whose levels are in the mid-range (20 ≤ *A* ≤ 50 dB SPL), as used in this MAA experiment, *γ*(*f*, *A*) is proportional to the stimulus level; that is, *γ*(*f*, *A*) = *A* · *γ*_0_(*f*), where *γ*_0_(*f*) is different for every fiber as determined by the location along the cochlear partition that the fiber innervates.

The function *B*(*f*) governs the synchronization of the fiber response which decreases with the increase of both frequency and the level of the simple tone stimuli. In this study we refer only to the dependence of the synchronization on frequency. The AN synchronization data [30, 54, 55] is commonly modelled by a sigmoid function of the form

(16) $$\begin{array}{c} B\left(f\right)=1.5\frac{e^{-\beta \cdot f}}{1+e^{-\beta \cdot f}}, \end{array}$$

where *β* is a constant that determines the loss of the fiber's synchrony as a function of frequency. We chose *β* = 10^−5^ which corresponds to a loss of synchrony at around 3 kHz [38, 54–56].

Since in a MAA experiment both ears are involved, the derivation of MAA will take into account those fibers from the right and left cochleae that are most sensitive to the stimulus frequency. We ignore all other fibers whose IRs are significantly reduced in comparison to the most sensitive fiber. Since the AN fibers are statistically independent [2], therefore the *d*′ theorem can be applied in order to obtain the MAA from *N* fibers:

(17) $$\begin{array}{c} {\left(d^{\prime }\right)}^{2}=\overset{N}{\underset{n=1}{\sum }}{\left(d_{n}^{\prime }\right)}^{2}, \end{array}$$

where *N* is the number of independent nerve fibers and *d*_*n*_′ is the *d*′ (see (7)) that was derived for the *n*th fiber. Since MAA is obtained when *d*′ = 1, this implies that

(18) MAA=std(θ^ ∣ θ∗) =1∑n=1NR(f){stdRn(θ^ ∣ θ∗)}−2+∑n=1NL(f){stdLn(θ^ ∣ θ∗)}−2,

where ${\text{std}}_{\text{R}}^{n}\left(\hat{\theta }|\theta^{*}\right)$ and ${\text{std}}_{\text{L}}^{n}\left(\hat{\theta }|\theta^{*}\right)$ are the standard deviations of the estimator as obtained by the right and left *n*th AN fibers, respectively, while *N*_R_(*f*) and *N*_L_(*f*)are the number of fibers of the right and left auditory nerve, respectively. When the optimal estimation is considered, the standard deviation is replaced by the correspondent CRLB (see (11)) or BLB (see (12)).

**Figure 3:**
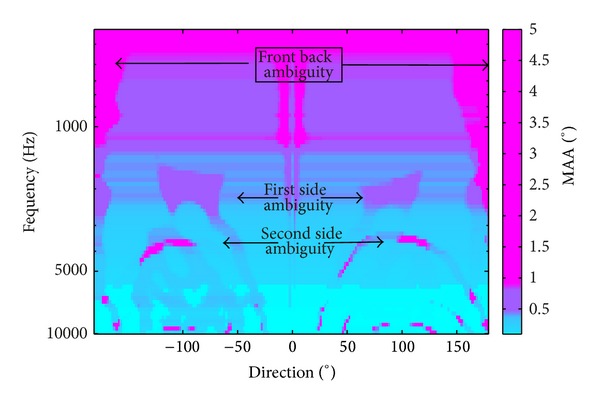
Prediction of MAA according to BLB derivation with a single ambiguity direction for all frequencies and directions based on the AN response (color online).

Figure 3 represents the prediction of MAA based on a BLB derivation with a single ambiguity (*L* = 1) as a function of both frequency and direction. The derivations were obtained by substituting (15) in (12). Equation (15) was derived for both right and left stimulations by using the correspondent HRIRs (see (2) that yields *λ*_R_(*t*, *θ*) and *λ*_L_(*t*, *θ*), the right and left auditory nerve instantaneous rates, respectively. In practice, only the fibers with a characteristic frequency equal to the stimulus frequency contribute to the MAA prediction. For the sake of simplicity, we chose *N*_R_(*f*) = *N*_L_(*f*) = *N*_0_; *γ*_0_(*f*) = 1 and *A* = 1. The number of fibers *N*_0_ was chosen so that CRLB at 500 Hz yielded MAA of 1°.

Throughout the frequency range, high values of MAA were obtained at the rear of the head (*θ* = 180°, *θ* = −180°). This is most likely due to front-back confusion. At approximately 2 kHz and its harmonics (4 and 8 kHz), relatively high values of MAA were obtained at approximately *θ* = 90° and *θ* = −90°. This most likely corresponds to the confusion between right and left. At directions that did not correspond to ambiguity, the values of the bound decreased with frequency.

**Figure 4:**
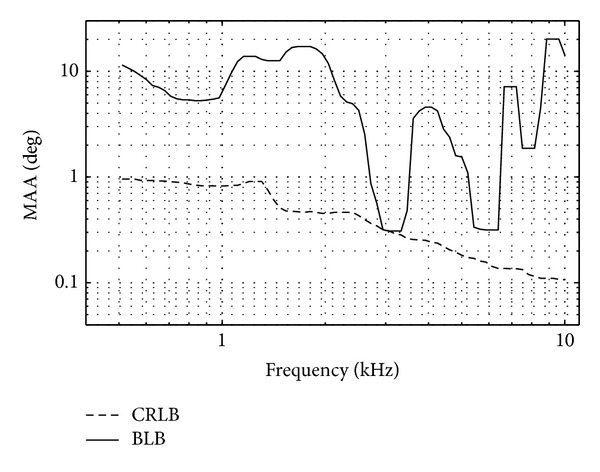
Normalized MAA according to CRLB and BLB as a function of frequency for a reference azimuth of 0° at AN.

Figure 4 represents the simulation results of MAA, derived by both lower bounds, CRLB and BLB, as a function of frequency when the reference direction was in front (θ* = 0). BLB was derived with at most 4 possible directions (*L* = 4). As can be expected, the estimated MAA according to BLB is greater than the CRLB estimates for all frequencies. At low frequencies, below 1 kHz, MAA according to BLB is about 10 times greater than the one yielded by CRLB. However, a more interesting difference between the two predictions is their dependence on frequency. CRLB derivation yielded a constant MAA of up to about 1 kHz and a monotonic decrease with increasing frequency for frequencies above 1 kHz. The BLB derivation yielded multiple peaks of MAA, in particular around 2, 4, 7, and 9 kHz.

The front-back confusion that exists throughout the whole frequency range is probably the reason for the difference in the MAA estimate according to the BLB and CRLB at low frequencies. Peaks at high frequencies (2, 4, 7, 9 kHz) can be attributed to the ambiguities that correspond to the similar phase obtained from tones coming from the sides or from the front of the head. According to the anthropometric data of the KEMAR dummy head [51], the head width is about 14 cm, which corresponds to a wavelength of tones with frequencies between 1.6 and 2.4 kHz [57]. These are the frequencies that yielded maximum MAA according to the BLB derivation.

While comparing the computational results of Figure 4 to the human performance shown in Figure 1, it seems that neither CRLB nor BLB is good predictors of human performance. By deriving MAA from CRLB, the dependence on frequency is totally different from data based on human performance. According to CRLB, MAA decreases monotonically as opposed to an oscillatory dependence in human experimental data. Although BLB reveals an oscillatory behaviour as a function of frequency, the predicted MAA has more oscillations as a function of frequency than human performance. In the next section we test whether this contradiction can be resolved by taking into account the binaural processing performed by CD cells in the brainstem nuclei such as SOC and IC.

## 5. MAA Prediction Based on the Superior Olivary Complex CD Cells

**Figure 5:**
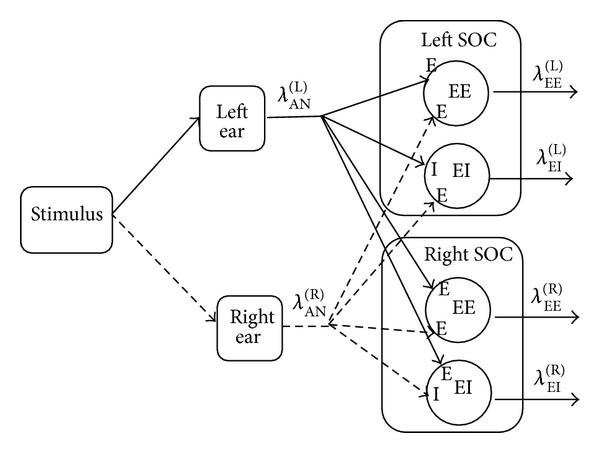
A schematic diagram of the binaural processing in the brainstem auditory pathway.

Figure 5 presents a schematic representation of part of the brainstem auditory pathway that is involved in binaural processing. The acoustic stimulus entering both ears innervates the auditory nerves. In Figure 5, the auditory nerves are represented by the left and right IRs, *λ*_AN_^(L)^ and *λ*_AN_^(R)^, respectively. The ANs stimulate both right and left SOCs. In each SOC, the two types of CD cells, EE and EI, are indicated.

Both EE and EI cells receive two independent inputs, one from each ear as Figure 5 indicates. Following [38], the output of both EE and EI cells is NHPP if the time interval (Δ) in which the two inputs can interact satisfies the condition Δ ≪ min⁡⁡{*τ*_R_, *τ*_L_}, where *τ*_R_ and *τ*_L_ are refractory periods of the right and left inputs.

The IR of the EE cells is obtained by

(19) λEE(t,θ)=λAN(L)(t,θ)∫t−ΔEEtλAN(R)(t′,θ)dt′+λAN(R)(t,θ)∫t−ΔEEtλAN(L)(t′,θ)dt′.

Since both right and left EE cells receive similar inputs, their output IRs are also identical; that is,

(20) $$\begin{array}{c} \lambda_{\text{EE}}^{\left(\text{R}\right)}\left(t,\theta \right)=\lambda_{\text{EE}}^{\left(\text{L}\right)}\left(t,\theta \right)=\lambda_{\text{EE}}^{}\left(t,\theta \right). \end{array}$$

A possible coincidence window length is Δ_EE_ = 20 *μ*sec [58]. The value of this length, which was previously used in theoretical models [24, 25, 29], satisfies the condition Δ_EE_ ≪ min⁡⁡{*τ*_R_, *τ*_L_}, since the refractory period at the auditory nerve is in the order of 500 *μ*sec to 1 m sec [59–62].

EI cells receive excitatory and inhibitory inputs. An EI in the right SOC (Figure 5) receives an excitatory input from the left side and an inhibitory input from the right side that yields

(21) $$\begin{array}{c} \lambda_{\text{EI}}^{\left(\text{R}\right)}\left(t,\alpha \right)=\lambda_{\text{L}}\left(t,\alpha \right)\left(1-\overset{t}{\underset{t-\Delta_{\text{EI}}}{\int }}\lambda_{\text{R}}\left(t^{\prime },\alpha \right)dt^{\prime }\right). \end{array}$$

On the other hand, an EI cell in the left SOC receives the antisymmetric inputs, that is, an excitatory input from the right side and an inhibitory input from the left side (Figure 5) that yields

(22) $$\begin{array}{c} \lambda_{\text{EI}}^{\left(\text{L}\right)}\left(t,\alpha \right)=\lambda_{\text{R}}\left(t,\alpha \right)\left[1-\overset{t}{\underset{t-\Delta_{\text{EI}}}{\int }}\lambda_{\text{L}}\left(t^{\prime },\alpha \right)dt^{\prime }\right]. \end{array}$$

A possible coincidence window length is Δ_EI_ = 200 *μ*sec [34]. This length is ten times longer than what was used in EE cells. However, it satisfies the condition Δ_EI_ < min⁡⁡{*τ*_R_, *τ*_L_}, which guarantees that EI cells behave as NHPP if their inputs also behave as NHPP [37].

In deriving MAA from the SOC CD cells, we assume that the outputs of the EE and EI cells are statistically independent. Therefore, MAA can be derived by using the *d*′ theorem (see (17)) which implies that

(23) MAA=std(θ^ ∣ θ∗)=(NEE(R)(f){stdEE(R)(θ^ ∣ θ∗)}2+NEE(L)(f){stdEE(L)(θ^ ∣ θ∗)}2   +NEI(R)(f){stdEI(R)(θ^ ∣ θ∗)}2   +NEI(L)(f){stdEI(L)(θ^ ∣ θ∗)}2)−1/2,

where $\text{st}{\text{d}}_{\text{EE}}^{\left(\text{R}\right)}\left(\hat{\theta }|\theta^{*}\right)$, $\text{st}{\text{d}}_{\text{EE}}^{\left(\text{L}\right)}\left(\hat{\theta }|\theta^{*}\right)$, $\text{st}{\text{d}}_{\text{EI}}^{\left(\text{R}\right)}\left(\hat{\theta }|\theta^{*}\right)$, and $\text{st}{\text{d}}_{\text{EI}}^{\left(\text{L}\right)}\left(\hat{\theta }|\theta^{*}\right)$ are the standard deviations of the estimator that were obtained by the right and left EEs and the right and left EI cells, respectively. The values *N*_EE_^(R)^(*f*), *N*_EE_^(L)^(*f*), *N*_EI_^(R)^(*f*), and *N*_EI_^(L)^(*f*) are the number of the right and left EE and EI cells at the SOC. When the optimal estimation is considered, the standard deviations are replaced by the correspondent lower bounds, CRLB (see (11)) or BLB with *L* = 4 (see (12)). The relevant instantaneous rates are obtained by substituting the EE IRs (see (19)) and the EI IRs (see (21) and (22)).

**Figure 6:**
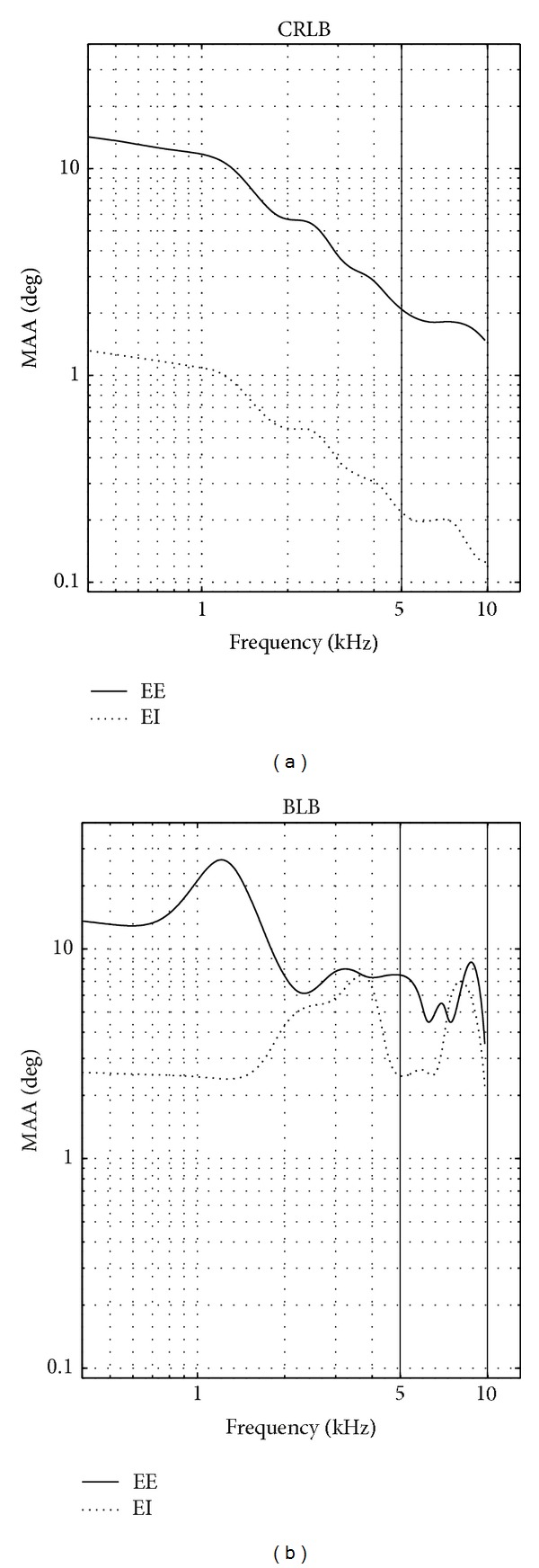
Predicted MAA according to CRLB (a) and BLB (b) as a function of frequency for a reference azimuth of 0° at the SOC level with either single EE cell or single EI cell.

In order to demonstrate the difference between the MAA derivations as obtained by EE and EI cells, we calculated (23) with either a single EE cell (*N*_EE_^(R)^(*f*) = *N*_EE_^(R)^(*f*) = 1; *N*_EI_^(R)^(*f*) = *N*_EI_^(L)^(*f*) = 0) or a single EI cell (*N*_EE_^(R)^(*f*) = *N*_EE_^(R)^(*f*) = 0; *N*_EI_^(R)^(*f*) = *N*_EI_^(L)^(*f*) = 1).  Figure 6 exhibits the resulting derivations as a function of frequency for a reference azimuth of 0°. According to CRLB, both EE and EI yielded a monotonic decrease as a function of frequency. But EE yielded a MAA with an order of magnitude greater than the one predicted from the EI response. On the other hand, the MAA that the EI yielded was similar to the one obtained by the AN response (Figure 4). One can then conclude that at the SOC level, EI processing caused minor information loss. However, due to EE processing some of the information that was included in the AN was lost.

When the MAA prediction was based on the BLB derivation (Figure 6(b)), both EE and EI yielded an oscillatory behavior as a function of frequency. When EE response was considered, the predicted MAA revealed local maxima at around 1.3 and 8 kHz, whereas the EI response yielded local maxima at 3.5 and 8 kHz.

**Figure 7:**
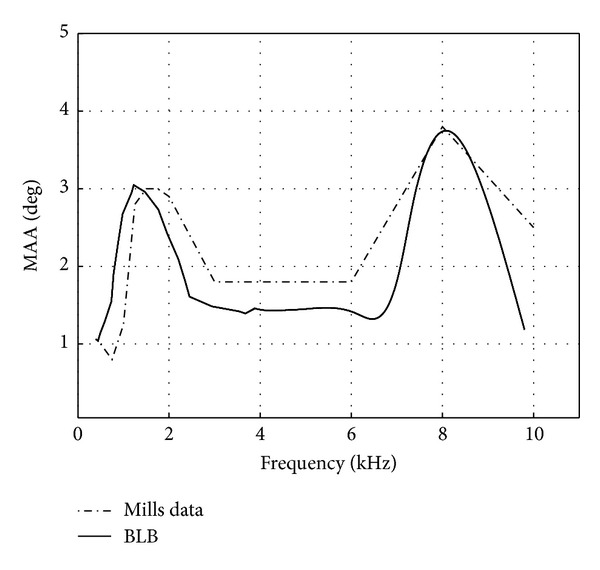
Predicted MAA according to BLB as a function of frequency for reference azimuth of 0° at the SOC level along with Mills' experimental data.

It is clear from Figure 6 that both EE and EI are required in order to match the experimental results (Figure 1).  Figure 7 represents the predicted MAA according to BLB with the following choice EE and EI cells:

(24) NEE(R)(f)=NEE(L)(f)={200f<1250 Hz251250≤f<40000f≥4000NEI(R)(f)=NEI(L)(f)={0f<40003f≥4000.

Note that (24) is consistent with physiological data indicating that EE cells mostly innervate signals with low frequencies, while EI cells are most sensitive to signals with high frequencies (e.g., [31, 32]). The predicted MAA has 2 peaks at about the same locations as the experimental data.

## 6. Discussion

The stochastic properties of neural spike trains in the auditory pathway were used in order to predict human performance in sound localization. We have shown that it is possible to predict human performance in an MAA experiment based on simple tones.

As in any JND experiment, predicting human performance was based on two main assumptions: (1) the brain is an unbiased optimal processor and (2) the neural spike trains behave as NHPP. The methodology involved deriving lower bounds based on the stochastic properties of neural spike trains [21, 22, 24, 25, 38].

When JND is predicted by deriving a lower bound, its significance is obtained by comparing it to experimental results as a function of a physical parameter. In this paper, we compared the bound prediction of MAA as a function of the stimulus frequency.

In an MAA experiment with simple tones, the information about the origin of the stimulus might be ambiguous at high frequencies. We have shown that the ambiguous interpretation of the HRTF phase data is probably the reason for the oscillatory behaviour of MAA as a function of frequency in human performance. This was demonstrated by the usage of two lower bounds, CRLB and BLB. In general, one can expect that the predictions of BLB will be greater than those obtained by CRLB. But the derivation demonstrated in this study reveals a totally different dependency on frequency. CRLB that took into account only the approximate true origin of the stimulus failed to predict oscillatory behavior. On the other hand, BLB, which considered ambiguity, succeeded in predicting the oscillatory behavior.

We further compared the predictions that were based on the AN outputs with those obtained by the SOC outputs. Although, both BLB predictions yielded an oscillatory behavior, it seems that the SOC output obtained a better prediction in respect to psychoacoustical data. When the AN output was considered, MAA local maxima were derived at frequencies 1.5, 2, 4, 7, and 9 kHz (Figure 4). When SOC was considered, some of the local maxima disappeared. It seems that loss of information due the SOC processing reduced the effect of the phase ambiguity.

The SOC outputs were derived by CD cells that processed the binaural information. The main task of CD cells is probably to extract binaural cues, with EE cells most likely extracting ITD and the EI cells extracting ILD [31, 32, 38]. Both ITD and ILD contribute to estimating the signal direction. In fact, our calculation of MAA, as derived by BLB, has shown that both EE and EI cells are required for predicting the experimental results.

We expect that the brain as an optimal system seeks to achieve a monotonic descending dependency of MAA as a function of frequency as predicted by CRLB. However, the physical constraints (i.e., the ambiguous phases) prevent the brain from achieving this goal.

When MAA was derived from the AN response or a single EI response, the predicted MAA according to BLB derivation was ten times greater than that predicted by CRLB at low frequencies (below 1.5 kHz). This difference was explained by the front-back confusion. However, this effect was almost eliminated when the EE response was considered. At low frequencies, the nerve response is synchronized to the stimulus; that is, *B*(*f*) → 1 in (16). Although the synchronization exists in the AN response, it was not sufficient for overcoming the front-back confusion. However, it played an important role at the SOC level where ITD was efficiently extracted [38]. Therefore, at the SOC level, at low frequencies, both lower bounds, CRLB and BLB, yielded almost the same MAA.

The methodology used in this study was to express the lower bounds by analytical expressions as derived in [21, 24, 25, 38]. This was possible, since we assumed that the neural spike trains behave as NHPP at AN. However, in using this assumption, the discharge history including the refractory period, which is a basic characteristic of every neuron [63–68], was ignored. Other models of neural spike trains that take into account the refractory period might be a better choice for describing the neural spike trains [66, 69–73]. Yet, the usage of the NHPP model and the outcome lower bounds approach has been successful in predicting several additional properties of the auditory system. Consider, for example, (1) the prediction of level and frequency discrimination as a function of the stimulus duration, level, and frequency [21, 24], and (2) ITD and ILD as a function of frequency [38]. In these studies it seems that the NHPP approach is adequate for describing steady-state responses of continuous stimuli [74–76].

This study aimed to show the potential in using lower bounds in order to predict human performance in psychoacoustical experiments and particularly the importance of considering ambiguous information. However, we do not claim that the simple model presented in this paper is the exact biological model. Further research is required in order to quantitatively predict human performances. Such studies should include a generalized cochlear model, a synapse model, and models of the brainstem nuclei.
